# Neuropeptide Y and Metabolism Syndrome: An Update on Perspectives of Clinical Therapeutic Intervention Strategies

**DOI:** 10.3389/fcell.2021.695623

**Published:** 2021-07-09

**Authors:** Yinqiong Huang, Xiahong Lin, Shu Lin

**Affiliations:** ^1^Department of Endocrinology, The Second Affiliated Hospital of Fujian Medical University, Quanzhou, China; ^2^Department of Endocrinology, The Seventh Affiliated Hospital of Sun Yat-sen University, Shenzhen, China; ^3^Centre of Neurological and Metabolic Research, The Second Affiliated Hospital of Fujian Medical University, Quanzhou, China; ^4^Diabetes and Metabolism Division, Garvan Institute of Medical Research, Darlinghurst, NSW, Australia

**Keywords:** neuropeptide Y, metabolic syndrome, arcuate nucleus, clinical intervention strategy perspective, hypothalamus

## Abstract

Through the past decade of research, the pathogenic mechanisms underlying metabolic syndrome have been suggested to involve not only the peripheral tissues, but also central metabolic regulation imbalances. The hypothalamus, and the arcuate nucleus in particular, is the control center for metabolic homeostasis and energy balance. Neuropeptide Y neurons are particularly abundantly expressed in the arcuate of the hypothalamus, where the blood-brain barrier is weak, such as to critically integrate peripheral metabolic signals with the brain center. Herein, focusing on metabolic syndrome, this manuscript aims to provide an overview of the regulatory effects of Neuropeptide Y on metabolic syndrome and discuss clinical intervention strategy perspectives for neurometabolic disease.

## Introduction

Metabolic syndrome (MS) is a disease characterized by obesity, dyslipidemia, high blood pressure, hyperglycemia, and insulin resistance. The increasing incidence of MS is a result of type 2 diabetes mellitus, cardiovascular disease (CVD), and other risk factors linked to early death (Spahis et al., 2017). MS is a cluster of major health risk factors associated with increased incidence of type 2 diabetes and CVD. Although the definition and criteria for MS vary, all definitions include important risk factors, such as a large circumference, elevated blood pressure, low high-density lipoprotein level, elevated levels of triglycerides, and hyperglycemia (Huang, 2009).

The pathogenic mechanisms underlying MS are complicated. In the past, these have mainly focused on peripheral tissues. However, in recent years, it has been found that central metabolic regulation imbalance may play an essential role. As the body’s metabolic regulation center, the hypothalamus receives peripheral metabolic information, integrates peripheral signals, and regulates energy homeostasis by controlling a series of neuroendocrine functions (Konner et al., 2009; Williams and Elmquist, 2012). In the arcuate (Arc) nucleus of the hypothalamus, there are two major populations of neurons that regulate energy balance. One group expresses orexigenic neuropeptides, including neuropeptide Y (NPY) and Agouti-related peptide (AgRP), while the other expresses anorexigenic proopiomelanocortin (POMC) and cocaine and amphetamine regulated transcripts (CART) (Gropp et al., 2005; Luquet et al., 2005). NPY neurons, located in the Arc of the hypothalamus where the blood-brain barrier is weak, are involved in the central regulation of metabolic homeostasis and energy balance. Therefore, they are the first-line neurons of the brain to integrate peripheral metabolic signals to regulate food intake and energy expenditure. Arc NPY neurons secrete NPY, AgRP, and the neurotransmitter GABA; as such, they are also called NPY-AgRP-GABA neurons. NPY/AgRP neurons are glucose inhibited neurons (Fioramonti et al., 2007), besides, they express insulin receptor (InsR) and leptin receptor (LepR), which can sense peripheral glucose and lipid metabolism signals. Arc NPY neurons project to other metabolic regulation-related nuclei, and thereafter through the autonomic nerve and endocrine system to centrally regulate metabolic balance. Arc NPY neuron response to peripheral metabolic alterations, increase food intake, decrease energy expenditure, and initiate ketogenesis. NPY is closely related to obesity and other metabolic diseases (Koch and Horvath, 2014). Therefore, assessing the metabolic regulation function of Arc NPY neurons and their relationship with the physiological status of metabolic diseases has clinical significance for the prevention and treatment of metabolic-related diseases.

## NPY System

Neuropeptide Y was isolated from porcine hypothalamus for the first time in 1982 (Tatemoto et al., 1982). NPY is a 36-amino acid peptide and has 70% homology with peptide YY (PYY) and a 50% homology with pancreatic polypeptide (PP). As such, the three are classified into the same NPY family. NPY is an abundant peptide in the mammalian brain. In the central nervous system, NPY is widely distributed in various areas of the brain, with higher concentrations in the Arc of the hypothalamus, the paraventricular (PVN) nucleus of the hypothalamus, the supraoptic nucleus, the superior nucleus, median eminence, dorsal medial hypothalamic (DMH) nucleus, paraventricular thalamic nucleus, amygdala, hippocampus, nucleus tractus solitarius (NTS), locus coeruleus, nucleus accumbens and cerebral cortex (Morris, 1989). NPY is also expressed in the peripheral sympathetic nervous system and is stored and released together with norepinephrine (Nozdrachev and Masliukov, 2011).

Besides the liver, heart, spleen, kidney, urogenital tract, and vascular endothelial cells also express NPY (Cavadas et al., 2001; Silva et al., 2005). The distribution of NPY receptors is also widespread. NPY binds to receptors to activate specific signaling pathways and produce biological effects. To date, five NPY receptors have been successfully cloned from mammals, namely Y1, Y2, Y4, Y5, and Y6 (Pedragosa-Badia et al., 2013), which all belong to the family of G protein-coupled receptors (Cabrele and Beck-Sickinger, 2000). In the human central and peripheral nervous system, neuropeptide Y receptors are encoded by different genes and have different tissue distribution and intracellular signaling pathways, thereby indicating that the NPY system is involved in a variety of physiological processes and plays different roles in different physiological processes. Y1 receptor is critical to feeding behavior. Studies have shown that selective NPY-Y1 and NPY Y5 receptor agonists strongly promote feeding behaviors (Bo et al., 2016). Furthermore, the increased expression of Y2 and Y4 receptors will produce anorexia effects. Y2 receptor knockout model was found to have weight gain, fat deposition and hyperorexia (Naveilhan et al., 1999). Y4 receptor knockout mice displayed a thin phenotype with lower body weight (Sainsbury et al., 2002).

## Interactions Pathways of NPY Neurons

Studies have documented the effect of central NPY on energy homeostasis, alongside the molecular mechanisms underlying these effects, including central and peripheral signaling pathways.

### Neural Mechanism of NPY Neurons Regulating Metabolism

**FIGURE 1:**
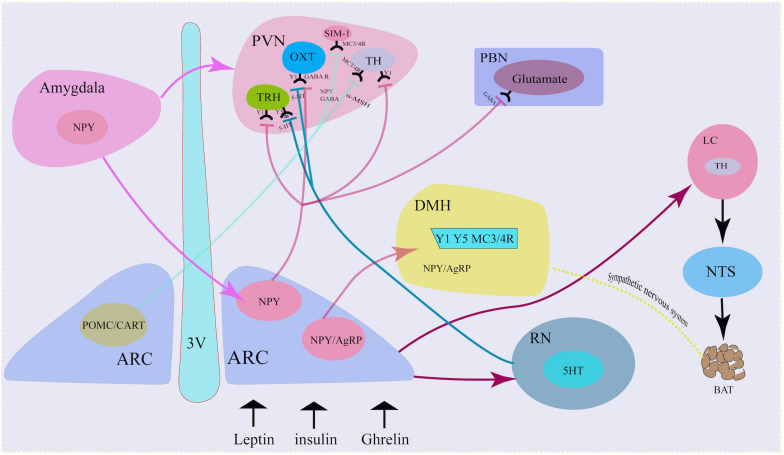
Neural mechanism of NPY neurons. NPY co-locate with neurotransmitters in the sympathetic nervous system. NPY in the Arc projected to the PVN *via* the activation of Y1 receptors on the TH neurons, TRH neurons and OXT neurons. Similarly, Arc NPY projected to DMH *via* Y1, Y5, and MC4R receptors. Besides hypothalamus, amygdala also secreted NPY and projected to Arc and PVN. 3 V, third ventricle; ARC, arcuate nucleus; TH, tyrosine hydroxylase; PVN, paraventricular nucleus; NTS, nucleus tractus solitarius; DMH, dorsomedial hypothalamus; LC, locus coeruleus; AgRP, agouti- related peptide; POMC, proopiomelanocortin; CART, cocaine- and amphetamine-regulated transcript; GABA, g-aminobutyric acid; a-MSH, a-melanocyte-stimulating hormone; OXT, oxytocin.

Central NPY neurons can integrate peripheral metabolic signals *via* neural pathways for regulating energy homeostasis (Figure 1).

#### NPY and Central Sympathetic Nervous System

Neuropeptide Y mainly functions as a sympathetic co-transmitter. NPY not only co-locates in neurons with other neurotransmitters (such as norepinephrine) but is also synthesized and released by non-neuronal cells. The traditional neurotransmitter norepinephrine and non-adrenergic transmitter adenosine triphosphate are stored in a large vesicle pool with small dense core vesicles, while NPY is stored in a small vesicle pool with large dense core vesicles, co-localized with norepinephrine (Merten and Beck-Sickinger, 2006). Although multiple neurotransmitters exist in the same vesicle, their release patterns are different due to the varying doses of neurotransmitters at the ends of axons and the activation of sympathetic nerves. For example, studies have shown that although adenosine triphosphate and norepinephrine co-locate in vesicles, the doses of adenosine triphosphate and norepinephrine in each vesicle may be uneven, due to the stimulation intensity of the sympathetic nervous system (Donoso et al., 2004; Ralevic, 2009). Adenosine triphosphate and norepinephrine are mainly released under low-frequency activation of the sympathetic nerve (Todorov et al., 1994), while NPY is released in response to strong, continuous and high-frequency activation of the sympathetic nerve.

#### Arc NPY Neuron-PVN Neural Pathway

Neuropeptide Y neurons projected to the PVN (Hill, 2012). The PVN is adjacent to the top of the third ventricle and is an important endocrine and metabolic regulation nucleus. Damage to PVN can lead to obesity. Single-minded-1 neuron (SIM-1) in PVN highly expresses melanocortin 3/4 receptor (MC3/4R), which is activated by α-melanocyte-stimulating hormone (α-MSH) and inhibits food intake. MC4R knockout can increase appetite and increase food intake. Activating the projection of PVN to the ventromedial nucleus of the hindbrain (VMH), the vagus nerve complex and locus coeruleus (LC) can also inhibit food intake. PVN neurons secrete corticotropin-releasing factor (CRF) and oxytocin (OXT), both of which inhibit food intake. NPY neuron-PVN is a classic neural pathway that integrate metabolic signals and to convey information from the ARC to other brain areas involved in appetite regulation and energy homeostasis (Simpson et al., 2008; Morton et al., 2014).

Neuropeptide Y/AgRP neurons release NPY to PVN neurons. At the same time, POMC neurons cleave and release α-MSH, an anorexigenic peptide that binds to and activates MC3/4R. α-MSH combines with MC3/4R in PVN neuron and functions to inhibit food intake, while NPY antagonizes the effect of α-MSH and increases food intake (Waterson and Horvath, 2015).

Arcuate NPY neurons project to PVN tyrosine hydroxylase (TH)-expressing neurons, reducing Y1 receptor-mediated TH expression, thereby reducing sympathetic activity and brown adipose tissue (BAT) thermogenesis and modulating energy expenditure. Increased NPY expression in the ARC under fasting, stress, or chronic-overfeeding conditions, acting *via* PVN Y1 receptors, results in the inhibition of TH tonus and sympathetically innervated BAT thermogenesis by downregulating uncoupling protein 1(UCP1) expression in BAT (Shi et al., 2013).

Arcuate NPY neurons also release NPY to PVN thyrotropin-releasing hormone (TRH) positive neurons under peripheral metabolic signals, such as ghrelin, and bind to Y1/Y5 receptors, inhibit cAMP-PKA signal, reduce TRH gene transcription, and reduce thyroid hormone release. If this process is dysfunctional, this will result in the inhibition of thyroid hormone release, which will indirectly decrease glucagon secretion and impair the function of pancreatic β cells and type 1 diabetes *via* the autonomic nervous system (Lechan and Fekete, 2006; Vella et al., 2011).

The NPY-PVN OXT projection is involved in the initiation of food intake. NPY neurons release GABA and NPY to OXT-positive neurons in PVN, activate GABA_*A*_ receptors and NPY-Y1-cAMP-PKA pathways, respectively, thereby inhibiting OXT neurons and reducing the release of oxytocin. This thereafter increases appetite, decreases basal metabolic rate and decreases bone synthesis rate (Atasoy et al., 2012).

Corticotropin-releasing factor neurons in the hypothalamic PVN initiate hypothalamic–pituitary–adrenal axis activity through the release of CRF into the portal system. The recent discovery of neurons expressing CRF receptor type 1 (CRFR1), the primary receptor for CRF, adjacent to CRF neurons within the PVN, suggests that CRF also signals within the hypothalamus to coordinate aspects of the stress response. CRFR1 neurons receive the majority of monosynaptic input from within the hypothalamus, mainly from the PVN itself. Locally, CRFR1 neurons make GABAergic synapses on parvocellular and magnocellular cells within the PVN. CRFR1 neurons resident in the PVN also make long-range glutamatergic synapses in autonomic nuclei such as the nucleus of the solitary tract (Jiang et al., 2018).

Neuropeptide Y neurons project mainly to second-order neurons in the PVN, but also to other encephalic region including the DMH, the lateral hypothalamus(LH) and the ventromedial hypothalamus(VMH) (Waterson and Horvath, 2015), which process the received metabolic information and project to multiple neurocircuits, and play an important role in maintaining energy homeostasis. Deletion of the DMH leads to a reduce of food intake and increase of energy expenditure, a hypophagic lean phenotype (Bellinger and Bernardis, 2002; Rezai-Zadeh et al., 2014). NPY is also expressed in neurons within the DMH. NPY in the DMH serve as an important neuromodulator for maintaining energy homeostasis. Knockdown of NPY expression in the DMH promoted development of brown adipocytes through the sympathetic nervous system, increased energy expenditure, reduced food intake, and thus prevented diet-induced obesity (Chao et al., 2011).

#### Arc NPY Neuron-Locus Coeruleus-NTS/Raphe Nucleus Projection

In response to fasting, Arc NPY neurons release NPY to the noradrenergic neurons in the locus coeruleus (LC) and PVN, and bind to the Y1 receptor to increase food intake. Arc NPY neurons can also project through the PVN-NTS, inhibit sympathetic preganglionic neurons, down-regulate brown fat UCP-1 expression, reduce body energy expenditure, and down-regulate the basal metabolic rate.

The raphe nucleus (RN) is rich in 5-hydroxytryptamine (5-HT) neurons, which can release 5-HT to NPY neurons *via* projection, bind to 5-HT1B receptors, and reduce NPY expression. On the other hand, the RN can also release 5-HT to PVN TRH and OXT neurons *via* its projections, combined with 5-HT2A receptors, and decrease the inhibitory effect of NPY on the electrical activity of PVN neurons, while promoting the release of hormones such as TRH and OXT, resulting in the inhibition of food intake and increase in the basal metabolic rate (Lechan and Fekete, 2006).

#### Central Amygdala NPY Neurons-Arc NPY Neuron Projection

Neuropeptide Y was not only produced in the hypothalamus, but also in extra-hypothalamic nuclei, such as the amygdala. Ip et al. found that under stress and a high-fat diet, NPY expression in both the central amygdala and Arc increased. Specific NPY overexpression in central amygdala NPY neurons project to the Arc and PVN-NPY neurons, resulting in increased food intake and decreased energy expenditure, and thus the development of obesity, which can be attenuated by ablation of the NPY in the central amygdala (Ip et al., 2019). NPY neurons in the central amygdala control feeding after stress combined with a high-fat diet by diminishing insulin signaling capacity (Ip et al., 2019).

#### Arc NPY Neuron-PVN-NTS Circuit

Arcuate NPY neurons receive nerve projections from the NTS, and thus integrate the gastrointestinal satiety signal from the vagus nerve to the center through the NTS to regulate feeding homeostasis. NTS’s A2 noradrenergic neurons can sense ghrelin signals, thereafter increasing the level of intracellular dopamine-β-hydroxylase. This enzyme catalyzes dopamine to demethyl norepinephrine (NE), increases neuron projections from these neurons to NPY neurons, and activates NPY P neurons, resulting in increased c-fos protein and neuron firing. This, in turn, causes an increase in food intake and a decrease in the basal metabolic rate. In addition, Arc NPY neurons release NPY to the PVN, through the PVN-NTS projection, indirectly activating medullary GABAergic neurons and NTS neurons, and ultimately inhibiting sympathetic preganglionic neurons, which increases food intake (Wu et al., 2012; Waterson and Horvath, 2015).

### Molecular Mechanism of NPY Neurons in Metabolism Regulation

**FIGURE 2:**
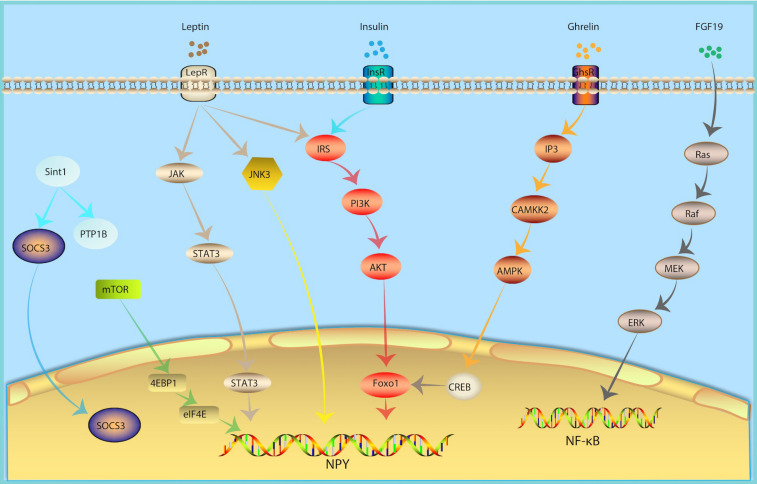
Molecular Mechanism of NPY Neurons in Metabolism Regulation. In response of peripheral metabolic changes, metabolic hormones including leptin, insulin and ghrelin regulated NPY signal *via* JAK-STAT3, PI3K-AKT-Foxo1, IP3-CAMKK2-AMPK pathway, respectively. FGF19, fibroblast growth factor-19; growth hormone secret agogue receptor (GHSR); InsR, insulin receptor; inositol triphosphate (IP3); JAK, Janus kinase; JNK, Jun NH2-terminal kinase; LepR, leptin receptor; mammalian target of rapamycin (mTOR).

In recent years, the mechanisms underlying the regulation of hypothalamic NPY neurons of glucose and lipid metabolism has been gradually discovered, revealing a series of cell signal pathways and related factors (Figure 2).

#### Leptin Signaling

Leptin is one of the metabolic hormones, which is correlated to body fat mass and energy balance (Woods and Seeley, 2000). In response to insufficient energy, levels of leptin are low. In response to excess energy, leptin secretion increases. A decrease in the plasma levels of leptin will lead to an increase in food intake (Weigle et al., 1997). Subsequently, the decline in leptin levels weakens the activation of POMC neurons and activates the NPY signal (Varela and Horvath, 2012b), consequently stimulating feeding behavior. The effects of leptin are mediated by LepR. LepR is expressed in multiple regions of the central nervous system, especially in NPY and POMC neurons in the Arc of hypothalamus (Cowley et al., 2001; Konner et al., 2007; Lin et al., 2010), which can integrate peripheral glucose and lipid metabolism signals. Arc NPY neurons are first-line neurons that sense leptin. NPY neuron-specific LepR knockout mice develop obesity and diabetes (Zhang et al., 2020), which is similar to that of db/db (LepR deficient mice) mice. POMC neurons are also first-line neurons that sense leptin. However, POMC neuron-specific LepR knockout mice do not have this same phenotype (Xu et al., 2018). Leptin knockout can relieve leptin’s inhibition of NPY neurons and cause obesity (Elias et al., 1999) and inhibit NPY neurons by chemogenetic tools which can improve food intake and blood glucose levels in diabetic mice (Zhang et al., 2019). The above evidence indicates that NPY neurons may be the main target of leptin. Leptin binding to the extracellular region of LepR activates Janus kinase (JAK). JAK binds to LepR and is phosphorylated, thereby activating STAT3. Once phosphorylated, STAT3 binds to the promoters of POMC and NPY to increase the expression of POMC and inhibit the expression of NPY (Vaisse et al., 1996; Mesaros et al., 2008; Ernst et al., 2009).

#### Insulin Signaling

Insulin is an important metabolic hormone for maintaining stable blood glucose and energy homeostasis. Insulin receptors were not only found in peripheral tissues, but also in the central nervous system, especially in NPY and POMC neurons of the Arc of hypothalamus (Cowley et al., 2001; Konner et al., 2007; Lin et al., 2010), which can integrate peripheral glucose and lipid metabolism signals. The insulin signaling pathways converge on PI3K. PI3K activates Akt. The activation of Akt leads to the phosphorylation and inactivation of the POMC expression inhibitor FoxO1, promoting the binding of STAT3 to the POMC promoter, and thereafter up-regulating the expression of POMC and downregulating the expression of NPY and AgRP (Myers and Olson, 2014). Eventually, this reduces food intake and increases insulin sensitivity.

#### Ghrelin Signaling

Ghrelin can activate the growth hormone secret agogue receptor (GHSR) in hypothalamic Arc NPY neurons, increase the level of inositol triphosphate (IP3) and promote the release of calcium from the endoplasmic reticulum. Calcium and calmodulin together activate CAMKK2, which phosphorylates AMPK’s alpha subunit N-terminal 172 threonine. Next, AMPK further phosphorylates ACC, decreases ACC activity, and reverse the ACC’s inhibitory effect on CPT1 and increases the expression of mitochondrial uncoupling protein 2 (UCP-2). Intracellular CREB is also phosphorylated, and pCREB and FOXO1 can up-regulate NPY/AgRP transcription, ultimately increasing peripheral fatty acid oxidation (Velasco et al., 2016).

#### NF-κB Signaling

The hypothalamus has the highest density of pro-inflammatory cytokine receptors (Hopkins and Rothwell, 1995). This inflammatory response in the hypothalamic may be the result of plasma cytokines entering the brain, given that some cytokines can cross the blood-brain barrier, including TNFα, IL-6, IL-1α, and IL-1β (Banks and Kastin, 1991; Gutierrez et al., 1993; Banks et al., 1994; Plotkin et al., 1996). These inflammatory cytokines play a role in the pathogenesis of anorexia (Moldawer et al., 1988; Schobitz et al., 1995). A high-fat diet can induce hypothalamus inflammation and damage the neural circuits that control feeding behaviors and energy homeostasis in pregnant and lactating mice, alongside affecting the Arc neurons formation in offspring, and an increase in NPY neurons (Lemes et al., 2018). PI3K, pAkt, and NF-κB levels were negatively correlated with the expression of NPY. Intracerebroventricular infusion of the PI3K inhibitor or NF-κB antisense could attenuate anorexia and reverse the expression of NPY (Hsieh et al., 2014). In addition, the activation of the IKKβ/NF-κB inflammatory pathway plays an important role in the process of POMC neurons loss, and POMC neurons play an important role in preventing obesity (Li et al., 2012; Thaler et al., 2012). Hypothalamic inflammation affects both NPY and POMC and promotes obesity.

#### mTOR Signaling

Mammalian target of rapamycin (mTOR) is a serine-theonine kinase as a sensor of energy balance, nutrients and oxygen (Roa and Tena-Sempere, 2010). mTOR is expressed in AgRP/NPY neurons in the ARC (Cota et al., 2006). Studies showed that hypothalmamic mTOR signaling plays a role in modulating energy balance responding to nutrient and hommone (Cota et al., 2006). Central administration of hormones, including ghrelin, leptin, thyroid hormone et al., induce phosphorylation of mTOR and activate its downstream targets, serine/threonine ribosomal protein S6 kinase (S6K1) and a ribosomal protein involved in translation S6 in the ARC, thus increase the expression of AgRP and NPY levels, increased food intake and increase body energy expenditure (Cota et al., 2008; Martins et al., 2012; Varela et al., 2012).

#### c-Jun NH2-Terminal Kinase 3

c-Jun NH2-terminal kinase (JNK) is a member of the MAPK family and an important metabolic stress signaling factor. JNK1, JNK2 and their isomers are widely expressed in various tissues and participate in the regulation of energy balance and insulin resistance. In contrast, JNK3 is expressed in neurons. JNK3 knockout mice showed a continuous high intake of high-fat diets, while they maintained normal food intake habits for normal chow. Hypothalamic LepR-positive NPY neuron-specific JNK3 knockout is a key factor leading to high-fat diet behavior, while LepR-positive POMC neuron-specific JNK3 knockout mice show no such feeding effect. In response to a high-fat diet, the absence of JNK3 signal in Arc NPY neurons will increase the electrical excitability of neurons, which increases food intake and causes obesity, suggesting that the Arc NPY neuron JNK3 signaling pathway plays an important role in maintaining metabolic homeostasis under conditions of metabolic stress such as that of a high-fat diet (Vernia et al., 2016).

#### Fibroblast Growth Factor 19

Fibroblast growth factor-19 (FGF19) is a hormone-like enterokine which can reduce the blood glucose of insulin resistance model mice (ob/ob mice and high-fat diet mice) and enhance insulin sensitivity. FGF19 can activate FGF receptors in Arc NPY neurons, which in turn activate the Ras-Raf-MEK-ERK1/2 signaling pathway and increases ERK1/2 phosphorylation. In contrast, inhibiting ERK1/2 phosphorylation can reduce blood glucose. The intraventricular injection of FGF19 can increase phosphorylated AKT levels in the liver, quadriceps, and white adipose tissue, suggesting that FGF19 can enhance insulin sensitivity, indicating that it may represent a potential drug for the treatment of type 2 diabetes (Marcelin et al., 2014).

## NPY and Metabolic Syndrome

### Energy Imbalance

The hypothalamus senses and integrates various signals from the blood and the third ventricle and regulates food intake (Kohno and Yada, 2012; Schneeberger et al., 2014). The arcuate nucleus is located in the ventromedial part of the hypothalamus, close to the median bulge which receives a rich blood supply. Therefore, information from peripheral tissues can easily enter the Arc region. In addition, they also receive the input of neural signals from multiple parts of the central nervous system (Dorr et al., 2015a; Sohn, 2015). The NPY system is a core component of the energy steady-state regulation system. NPY/AgRP neurons and POMC neurons, a group of antagonistic neurons, not only regulate the feeding process, but also participate in the subsequent coordination of the conversion, storage and utilization of carbohydrates and lipids (Varela and Horvath, 2012a). As such, NPY/AgRP neurons are seen as an important bridge for the center to participate in the distribution of peripheral nutrients. NPY/AgRP signaling in adult mice is very important for maintaining normal lipid and glucose homeostasis in peripheral tissues (such as liver, muscle, and pancreas). Depletion of NPY/AgRP neurons in newborn mice will affect the balance of lipid and carbohydrate metabolism. Mice lacking NPY neurons develop obesity and hyperinsulinemia in response to a normal chow diet, but exhibit reduced weight gain and abnormal improvement in glucose tolerance under a high-fat diet (Joly-Amado et al., 2012; Vahatalo et al., 2015), pointing to the role of NPY/AgRP neurons in coordinating nutrient distribution.

Although it is known that AgRP co-localize with NPY neurons in the Arc and AgRP neurons express NPY, there remains a significant sub-population of Arc NPY neurons that do not co-express AgRP (Luquet et al., 2005). A specific deletion of InsRs in AgRP neurons was not found to result in the same metabolic phenotype as NPY specific deletion (Konner et al., 2007; Loh et al., 2017), suggesting that non-AgRP expressing NPY neurons play a more important role in the regulation of energy homeostasis. The difference between non-AgRP NPY neurons and AgRP/NPY co-expression neurons has not yet been completely elucidated. Studies suggest that non-AgRP NPY neurons may be more critical in mediating insulin’s effects on food intake, energy expenditure, and peripheral insulin release, while AgRP/NPY co-expression neurons may be more essential in regulating hepatic glucose production (Sainsbury et al., 1997; Konner et al., 2007; Loh et al., 2017).

### Glucose Intolerance

Insulin plays a key role in the short-term and long-term control of blood glucose and energy homeostasis (Saltiel and Kahn, 2001; Plum et al., 2006; Loh et al., 2017). InsRs are also expressed in the hypothalamic nucleus. In POMC/CART neurons, insulin is considered to promote satiety, while in NPY/AgRP neurons, insulin signaling mainly inhibits food intake and increases energy expenditure. Studies have shown that the acute increase in insulin levels by direct administration can reduce the level of NPY in the arcuate nucleus of the hypothalamus (Schwartz et al., 1992; Benoit et al., 2002). In rodent models with an insulin deficiency, such as streptozotocin-induced diabetic rats, the levels of NPY in the hypothalamus were significantly increased, confirming the inhibitory effect of peripheral insulin on central NPY (Gelling et al., 2006). Interestingly, under obesity and insulin resistance, a long-term increase in insulin levels can also lead to an increase in hypothalamic NPY levels, indicating that hypothalamic NPY is resistant to the feedback regulation of insulin elevation. By inhibiting the hypothalamic NPY signaling pathway, insulin secretion will be increased (Konner and Bruning, 2012; Loh et al., 2015), thus participating in the pathogenesis of type 2 diabetes.

### Hypertension

In previous studies, it has been proved that NPY is related to various cardiovascular diseases, such as myocardial infarction, heart failure, and cardiac hypertrophy (Thulin and Erlinge, 1995; McDermott and Bell, 2007; Herring et al., 2019). NPY is the most abundant neuropeptide found in the heart, and was the first neuropeptide found in the heart identified in intramural sympathetic nerves closely related to coronary vessels. NPY is a strong vasoconstrictor. It was found that NPY has a positive time-changing and inotropic effect on the heart (Maisel et al., 1989; Millar et al., 1991). In peripheral cardiovascular tissues, NPY is often released together with norepinephrine, especially when sympathetic nerve activity increases. In addition, in the plasma of patients with hypertension, the level of NPY is elevated (Morris et al., 1997; Dorr et al., 2015b). In rats, chronic stress can double plasma NPY levels, increase blood pressure and promote the infiltration of macrophages in the intima, further leading to carotid artery occlusion (Li et al., 2005; Sun et al., 2017). Various lines of evidence indicate that NPY is involved in the pathological process of hypertension. In humans and animal models of hypertension, peripheral and central neuropeptide Y is related to the pathological mechanisms underlying hypertension (Ruohonen et al., 2009; Tan et al., 2018; Liu et al., 2020).

### Atherosclerosis

**FIGURE 3:**
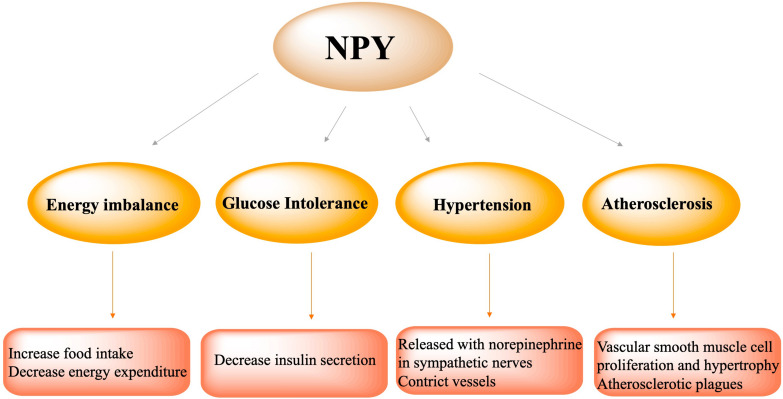
NPY participated in the pathophysiological process of metabolic syndrome, including energy imbalance *via* increasing food intake and decreasing energy expenditure, glucose intolerance *via* decreasing insulin secretion, hypertension *via* NPY release with norepinephrine in sympathetic nerves and vasoconstriction, atherosclerosis *via* vascular smooth muscle cell proliferation and hypertrophy and formation of atherosclerotic plagues.

In the cardiovascular system, NPY is expressed in coronary arteries, endothelial cells and cardiomyocytes innervated by sympathetic nerves. Endothelial cells are the barrier between the blood vessel wall and the blood. Endothelial dysfunction can lead to the proliferation and angiogenesis of lipids, inflammatory cells, blood clotting substances, and vascular smooth muscle cells, alongside promoting atherosclerotic plaques. Studies have shown that NPY-Y1, Y2, and Y5 receptors are expressed on endothelial cells, which are involved in the formation of atherosclerotic plagues. Under pathological conditions, NPY induces vasoconstriction and stimulates vascular smooth muscle cell proliferation and hypertrophy *via* the Y1R. Foam cells and chronic inflammation are critical in atherosclerosis. Studies have shown that the expression of NPY in atherosclerotic plaques significantly increases, and a large number of macrophages aggregate into foam cells and participate in the formation of atherosclerotic plaques *via* NPY-Y1, Y2 and Y5 receptors. In the process of atherosclerosis, the pathophysiological changes of vascular smooth muscle cells are extremely important. NPY can increase the levels of calcium in the cytoplasm of vascular smooth muscle cells, activate protein kinase C and promote mitosis, thereby leading to atherosclerosis. Hence, the down-regulation of NPY and its receptors can induce anti-atherosclerotic effects. Wu WQ et al. found that physical exercise increased plague collagen and smooth muscle cell content and decreased plaque lipid and macrophage content in apolipoprotein E-deficient mice by decreasing the expression of NPY and NPY Y1 receptors in vasculature, suggesting that the down-regulation of NPY and NPY receptors expression in the aorta is critical to the anti-atherosclerotic effect (Wu et al., 2019; Figure 3).

### Clinical Therapeutic Intervention Strategy

**FIGURE 4:**
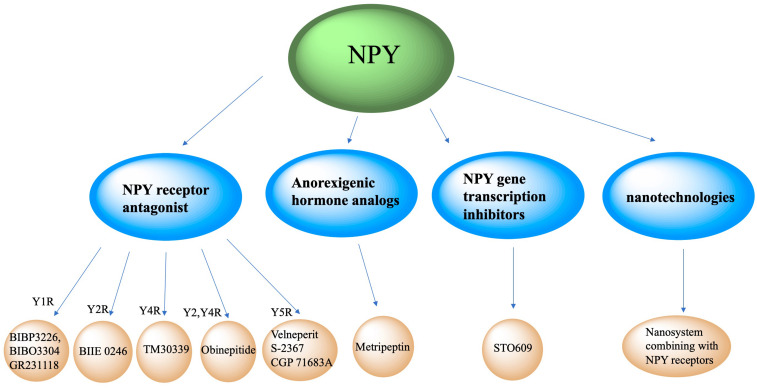
Clinical therapeutic intervention strategy of NPY. Intervention strategies targeting NPY include NPY receptor antagonists, anorexigenic hormones analogs, NPY gene transcription inhibitors and new technologies like nanosystem.

Neuropeptide Y neurons play critical role in metabolic diseases, including obesity, glucose tolerance, hypertension and atherosclerosis. Inhibiting NPY neuronal activity and antagonizing NPY expression may be important potential methods to regulate metabolism. Currently, intervention strategies targeting NPY may include NPY receptor antagonists, anorexigenic hormones (such as leptin) analogs, and NPY gene transcription inhibitors (Figure 4).

### NPY Receptor Antagonist

Neuropeptide Y neurons secrete NPY, which interact with POMC neurons, parabrachial nucleus and paraventricular nucleus neurons, and binds to its Y1-Y5 receptors to increase appetite. Antagonizing NPY receptors and blocking the NPY signaling pathway can attenuate obesity. The Y1 receptor is a 36-amino acid, and its binding affinity to NPY and PYY is much higher than that of PP. A recent published paper mentioned about specific target NPY Y1 receptor antagonism in improving insulin resistance and protects against diet induced obesity (Yan et al., 2021). Selective Y1 receptor antagonists include BIBP3226, BIBO3304 and GR231118. The Y2 receptor consists of 381 amino acids and binds NPY and PYY with a higher affinity than PP, has alongside having a high affinity for C-terminal fragments (Lafferty et al., 2020). BIIE 0246 is a selective antagonist of the Y2 receptor (Ueda et al., 2021). The Y4 receptor consists of 375 amino acids, and has a higher affinity for PP than PYY and NPY (Sun et al., 2017). TM30339, a pancreatic peptide analog, selectively antagonizes the Y4 receptor of NPY. Obinepitide is an analog of pancreatic peptide and peptide tyrosine (PYY3-36) which can selectively antagonize the Y2 and Y4 receptors of NPY, and thus avoids cardiovascular side effects caused by antagonizing the Y1 receptor. The Y5 receptor is a protein consisting of 445 amino acids. NPY, PYY, and PP can all be recognized and bound as ligands. Velneperit S-2367 and CGP 71683A are Y5 receptor selective antagonists. Selectively stimulating or blocking NPY receptors has therapeutic potential in the treatment of obesity and metabolic disorders (Lin et al., 2006).

### Anorexigenic Hormone Analogs

Neuropeptide Y neurons express anorexigenic hormone (such as leptin) receptors. When the receptors are activated, the expression of NPY is down-regulated to decrease appetite and food intake. Metripeptin, a leptin analog, can specifically bind to leptin receptors and activate the leptin signaling pathway of NPY neurons, which can significantly reduce appetite and promote fat metabolism (Baykal et al., 2020). The drug was approved for marketing by the US FDA in 2014.

### NPY Gene Transcription Inhibitors

This class of drugs inhibit NPY gene transcription in NPY neurons. STO609 can significantly inhibit the intracellular CaMKK2 activity of NPY neurons and reduce the phosphorylation of ACC by CaMKK2, expression of mitochondrial UCP-2, and p-CREB levels, thereby decreasing NPY transcription, decreasing appetite, and increasing basal metabolic rate (Anderson et al., 2008).

### New Clinical Therapeutic Intervention Prospect Involving Nanosystem

New technologies, such as nanotechnologies, may also be applied to NPY-related clinical therapeutic intervention strategies. Jiang ZQ et al. conjugated NPY Y1 receptor ligand (Asn6, Pro34)-NPY on the surface of doxorubicin-encapsulated nano Zeolitic imidazole frameworks-90, resulting in a more effective and faster targeted delivery and dual responsive release of DOX, significantly improving therapeutic efficacy in tumors and survival rates (Jiang et al., 2019). Another study created Y1 receptor ligand (NPY)-modified bubbles, finding that, compared with modified bubbles without NPY targeting, NPY-modified bubbles group showed a high tumor suppression effect and a prolonged survival time in Y1 receptor-overexpressed breast cancer treatments (Qian et al., 2020). Although nanosystems combining with NPY receptors are currently focused on tumor treatments, we believe nanotechnologies combined with NPY receptor ligands may play a role in neuroendocrine disease treatment.

## Conclusion

The universality and diversity of the distribution of NPY and its receptors enable the NPY system to participate in the regulation of multiple physiological processes, especially in the regulation of energy metabolism balance, alongside integrating information with peripheral hormones such as insulin, leptin, and the sympathetic nervous system. A number of complex regulatory circuits are used to precisely regulate energy metabolism. When the balance is disrupted, or the hypothalamus is affected by inflammatory factors, metabolic disorders will emerge, alongside manifestations of abnormal lipid metabolism, in the form of obesity, dyslipidemia and abnormal glucose metabolism such as high blood glucose and insulin resistance development. The NPY system plays an important role in the pathological mechanisms underlying MS, in which multiple receptors and metabolic pathways can be used as potential targets for drug development. Therefore, further research is warranted to thoroughly clarify the processes and interactions of various types of regulatory mechanisms in the NPY system.

Therefore, NPY and its receptors provide a significant prospect for the clinical intervention strategy of MS and drug development in the future. The intervention targeting NPY receptor with pharmaceuticals showed benefits to metabolic syndrome, however, the strategy is non-specific, and will cause other non-specific side effects, which should be paid attention to.

Therefore, it is necessary to select more precise and individualized intervention methods, precise treatment, and individualized treatment; specifically determine the indications of each strategy. Besides, together with the pharmaceuticals treatment of MS, exercice can reduce NPY and improve NPY-related target treatments, suggesting that encouraging patients to do exercise is quite important for treatment too.

Besides, nanosystems combined with NPY receptor ligands or new molecular biology therapy, which can achieve more precise targeted therapy might represent a potential method in the context of neuroendocrine disease treatment.

## Author Contributions

YH contributed in the literature search and manuscript writing. XL made the figures and the manuscript writing. SL contributed in the study design and manuscript writing. All the authors read and approved the final manuscript.

## Conflict of Interest

The authors declare that the research was conducted in the absence of any commercial or financial relationships that could be construed as a potential conflict of interest.

## Glossary

Arc: arcuate nucleus
BAT: brown adipose tissue
BDNF: brain-derived neurotrophic factor
CVD: cardiovascular disease
CART: cocaine and amphetamine regulated transcripts
CRF: corticotropin-releasing factor
CRFR1: corticotropin-releasing factor receptor type 1
DMH: dorsal medial hypothalamic
FGF19: fibroblast growth factor-19
Gpr17: G protein-coupled receptor 17
5-HT: 5-hydroxytryptamine
InsR: insulin receptor
JAK: Janus kinase
JNK: Jun NH2-terminal kinase
LepR: leptin receptor
LC: locus coeruleus
NE: norepinephrine
MS: metabolic syndrome
MC3/4R: melanocortin 3/4 receptor
α-MSH: α-melanocyte-stimulating hormone
NPY: neuropeptide Y
NTS: nucleus tractus solitarius
OXT: oxytocin
POMC: proopiomelanocortin
PYY: peptide YY
PP: pancreatic polypeptide
PVN: paraventricular
POA: preoptic area
RN: raphe nucleus
SIM-1: single-minded-1 neuron
TH: tyrosine hydroxylase
TRH: thyrotropin-releasing hormone
TrkB: tropomyosin receptor kinase B
UCP1: uncoupling protein 1
VMH: ventromedial nucleus of the hindbrain.
